# Effects of partial replacement of sugarcane silage with *Moringa oleifera* silage on carcass traits and meat quality of lambs

**DOI:** 10.1007/s11250-026-05309-x

**Published:** 2026-09-03

**Authors:** Gustavo Delmilho, Adibe Luiz Abdalla, Natana Mendes Marques, Theyson Duarte Maranhão, Vagner Ovani da Silva, Lumena Souza Takahashi, Leticia dos Santos Menezes, Mauro Sartori Bueno, Rafael Silvio Bonilha Pinheiro, Márcia Mayumi Harada Haguiwara, Ricardo Lopes Dias da Costa

**Affiliations:** 1 Center for Nuclear Energy in Agriculture (CENA), University of São Paulo (USP), Piracicaba, São Paulo Brazil; 2 Animal Science Institute (APTA/SAA), Nova Odessa, São Paulo, Brazil; 3 Department of Animal Production and Veterinary Medicine, Faculty of Veterinary Medicine and Animal Science, São Paulo State University (UNESP), Botucatu, São Paulo Brazil; 4 Institute of Food Technology, Meat Technology Center, Campinas, São Paulo Brazil; 5 Center of Carbon Research in Tropical Agriculture (CCarbon), University of São Paulo (USP), Piracicaba, São Paulo Brazil

**Keywords:** Alternative forage, Cooking loss, Feedlot, Oxidative stability, Small ruminants, Tropical nutrition

## Abstract

This study evaluated the effects of partially replacing sugarcane silage with *Moringa oleifera* silage on carcass traits and meat quality of lambs. Sixteen male Santa Inês × Dorper lambs (23.0 ± 2.00 kg; 6 months old) were assigned to either sugarcane silage (C) or a mixed silage containing sugarcane and *M. oleifera* (C + M; 50:50 on a dry matter basis) over a 75-day experimental period, including 15 days of adaptation and 60 days of evaluation. Carcasses were evaluated for fasting weight, hot and cold carcass weights, carcass yields, non-carcass components, fat cover, ribeye area, and commercial cut yields. Meat quality was assessed in the *Longissimus lumborum* muscle for pH, lightness (L*), redness (a*), yellowness (b*), chroma (C*), hue angle (h°), cooking loss, shear force, proximate composition, and oxidative stability based on thiobarbituric acid reactive substances (TBARS). Lambs fed the C + M diet had lower fasting and hot carcass weights than those fed the control diet (*p* < 0.01), whereas fat cover and ribeye area were not significantly affected. Meat dry matter, crude protein, ether extract, and ash contents did not differ between treatments (*p* > 0.05). Color parameters, oxidative stability, and shear force were also unaffected by dietary treatment. Cooking loss was higher in the C + M group (*p* < 0.05), indicating lower water-holding capacity under the evaluated conditions. Overall, replacing 50% of sugarcane silage with *M. oleifera* silage preserved most physicochemical meat quality attributes, although it reduced fasting and hot carcass weights and increased cooking loss. These findings indicate that *M. oleifera* silage may be used as an alternative forage resource in tropical lamb finishing systems, provided that its effects on carcass weight and water-holding capacity are considered.

## Introduction

Sheep meat production has gained relevance worldwide due to the adaptability of small ruminants and the increasing demand for high-quality lamb products (Santos-Silva et al. 2019). Improving growth efficiency while maintaining desirable carcass and meat quality traits is a key objective in modern sheep production systems, as feed costs represent a substantial proportion of lamb-finishing expenses (Antas-Urbano et al. 2015; Chacko et al. 2024). Therefore, nutritional strategies that support efficient tissue deposition without compromising product quality are essential for sustainable lamb production.

In tropical and subtropical regions, seasonal fluctuations in forage availability often require the use of conserved feeds during dry periods. Sugarcane is widely used as a forage resource because of its high biomass yield, agronomic adaptability, and availability in tropical production systems (Siqueira et al. 2012). However, sugarcane silage presents nutritional and fermentative limitations, including low crude protein concentration, alcoholic fermentation, and dry matter losses during ensiling (Jacovaci et al. 2017; Souza et al. 2022). These limitations have increased interest in combining sugarcane with alternative forages, rich in protein, capable of improving silage nutritional quality.

*Moringa oleifera* has emerged as a promising forage resource due to its high protein concentration, rapid growth, drought tolerance, and high biomass production (Gualberto et al. 2014; Benhammouche et al. 2021). In addition to its nutritional value, moringa contains bioactive compounds, including phenolic compounds, tannins, and saponins, which may influence oxidative processes and product quality. Recent in vivo research using sugarcane- and moringa-based silages in lamb diets reported changes in nutrient supply, feed intake dynamics, physiological responses, rumen microbial populations, and gaseous emissions (Delmilho et al. 2026). The consequences of feeding strategies, using moringa for carcass traits and the physicochemical quality of lamb meat, are still very necessary.

We hypothesized that the partial replacement of sugarcane silage with moringa silage would improve the nutritional composition of the forage while preserving carcass and meat quality attributes. Therefore, this study aimed to evaluate the effects of partially replacing sugarcane silage with moringa silage, on carcass traits and the physicochemical characteristics of lamb meat.

## Materials and methods

### Experimental design and animals

The experiment was conducted at the Animal Science Institute (APTA/SAA), Nova Odessa, São Paulo, Brazil. All animal procedures were approved by the institutional Animal Care and Use Committees (CEUA/IZ 346–2022; CEUA/CENA-USP 011/2016) and complied with national guidelines for the ethical use of animals in research. Sixteen intact male Santa Inês × Dorper lambs (6 months of age; 23.0 ± 2.00 kg initial body weight) were individually identified and randomly assigned to one of two dietary treatments (*n* = 8 per treatment) in a completely randomized design. Each animal was considered an experimental unit.

Animals were maintained under confinement conditions for 75 days, comprising 15 days of adaptation and 60 days of feeding and data collection. The dietary treatments consisted of: (1) control diet (C), containing sugarcane silage; and (2) C + M diet, containing a mixed silage composed of sugarcane and moringa at a 50:50 ratio on a dry matter basis. The chemical composition of the experimental silages, commercial concentrate, and raw forage ingredients is presented in Table 1. Dry matter, crude protein, ether extract, ash, and crude fiber were determined according to AOAC (2016), whereas neutral detergent fiber and acid detergent fiber were determined following Van Soest et al. (1991).

Moringa plants were cultivated at the institute’s experimental farm, harvested at the vegetative stage, manually chopped, and immediately ensiled in 25-kg plastic bags. Whole moringa plants were used for silage preparation. Sugarcane was obtained from a local supplier in Nova Odessa, São Paulo, Brazil, chopped, and ensiled following the same procedure. Both diets included a commercial concentrate and were formulated at a forage-to-concentrate ratio of 60:40 on a dry matter basis.

Feed was offered twice daily, at approximately 08:00 and 14:00 h. Refusals were collected and weighed before each subsequent feeding. Animals had free access to clean drinking water throughout the experimental period. Individual feed intake was monitored using an electronic feeding system (Intergado^®^, Brazil), which electronically identified each lamb and recorded changes in feed weight during feeder visits, allowing individual intake to be monitored throughout the trial.

### Carcass procedure and evaluation

At the end of the 60-day feeding and evaluation period, lambs were slaughtered in accordance with the Regulation for the Industrial and Sanitary Inspection of Animal Products (Silva and Komuro, 2024). Animals were subjected to approximately 16 h of feed withdrawal and subsequently weighed to obtain fasting body weight (FW). Slaughter was performed by captive-bolt stunning followed by exsanguination, skinning, and evisceration. Non-carcass components, including internal organs and external components, were individually weighed as described by Pillmore et al. (2024).

Hot carcass weight (HCW) was recorded immediately after evisceration. Carcasses were then chilled at 4 °C for 24 h, after which cold carcass weight (CCW) was recorded. Chilling loss was calculated from HCW and CCW. Hot carcass yield (HCY), cold carcass yield (CCY), and total non-carcass component weight (NCarc) were determined according to Vasconcelos et al. (2024). Carcass conformation and fat cover were visually assessed using a 1–5 scoring system.

Commercial cuts, including the leg, loin, rack, shoulder, breast, and neck, were obtained from the left half-carcass and individually weighed. A cross-sectional cut was performed between the 12th and 13th ribs to expose the *Longissimus lumborum* muscle. Ribeye area was subsequently determined following Gurgueira et al. (2022), and muscle samples were collected for meat quality analyses.

### Meat quality analyses

Meat pH and temperature were measured at 45 min (pH₄₅, T₄₅) and 24 h (pH₂₄, T₂₄) postmortem in the *semimembranosus* muscle using a portable digital pH meter with temperature compensation (HI 9126, Hanna Instruments^®^, USA). The instrument was calibrated using standard buffers (pH 4.01 and 7.01).

Meat color was evaluated using a portable colorimeter (Delta Vista d8, Delta Color). Color coordinates were expressed in the CIELAB system as L* (lightness), a* (red/green), and b* (yellow/blue), and Chroma (C*) and hue angle (h°) were calculated according to Lima et al. (2021).

Cooking loss was determined using standardized *Longissimus* steaks (2.5 cm thickness) cooked to an internal temperature of 70 °C. Warner/Bratzler shear force was measured using a TA-XT2i texture analyzer (Stable Micro Systems, UK), following Silva et al. (2025)

Proximate composition (dry matter, crude protein, ether extract, and ash) of *Longissimus lumborum* samples was determined according to Murariu (2023). Oxidative stability was assessed by thiobarbituric acid reactive substances (TBARS), peroxide index, and discoloration assays as described by Oancea et al. (2022).

### Statistical analysis

The experiment followed a completely randomized design with two dietary treatments (C: sugarcane silage; C + M: sugarcane + *Moringa oleifera* silage) and 16 lambs (*n* = 8 per treatment), with the individual animal considered the experimental unit. Statistical analyses were performed using SAS Studio (SAS Institute Inc., Cary, NC, USA). Treatment effects were evaluated by analysis of variance (ANOVA) using PROC GLM according to the model: $\:{Y}_{ij}=\mu\:+{T}_{i}+{\epsilon\:}_{ij}$, where $\:{Y}_{ij}$is the observation of animal * j*receiving treatment * i*, $\:\mu\:$is the overall mean, $\:{T}_{i}$is the fixed effect of diet, and $\:{\epsilon\:}_{ij}$is the residual error.

Residual normality and homogeneity of variances were assessed using the Shapiro–Wilk and Levene tests, respectively. When assumptions were not met, Welch’s t-test or the Wilcoxon rank-sum test was applied. Variables measured at different postmortem times were analyzed separately for each time point. Statistical significance was declared at *p* < 0.05, and results are presented as means ± standard deviation.

## Results

### Feeding behavior

**Fig. 1:**
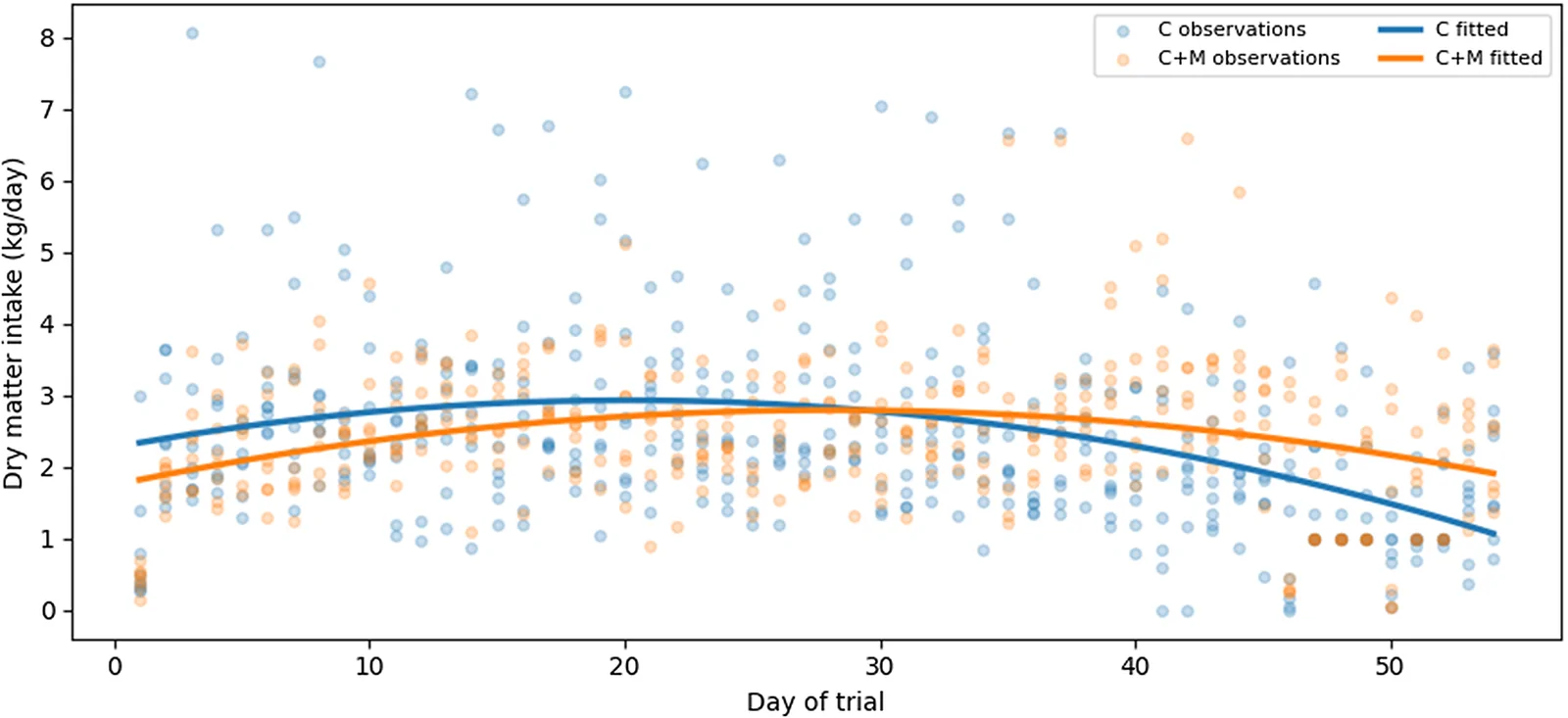
Daily dry matter intake (DMI) of lambs fed sugarcane silage (C) or sugarcane silage partially replaced by *Moringa oleifera* (C + M). Points represent individual observations, and lines represent mixed-model fitted curves

Dry matter intake (DMI) followed a significant curvilinear pattern throughout the 60-day evaluation period (*p* < 0.001; Fig. 1). Overall mean DMI did not differ between treatments (*p* = 0.891). However, a significant treatment × day interaction was detected (*p* = 0.001), indicating that intake trajectories differed between diets over time. DMI declined toward the end of the evaluation period in both treatments, although this reduction was less pronounced in lambs fed the C + M diet than in those who fed the control diet (C).

### Carcass characteristics

Lambs fed the C + M diet had lower fasting body weight and hot carcass weight than those fed the control diet (*p* < 0.05; Table 2). Cold carcass weight was numerically lower in the C + M group, with mean values of 16.8 and 14.7 kg for the C and C + M treatments, respectively, although the difference was not statistically significant (*p* = 0.06). No differences were observed between treatments in pH measured at 45 min and 24 h postmortem, carcass temperature, carcass conformation, or fat cover (*p* > 0.05). Overall, dietary treatment affected fasting body weight and hot carcass weight but did not significantly alter the evaluated qualitative carcass traits.

### Organs and non-carcass components

Rumen and intestinal weights were lower in lambs fed the C + M diet than in those fed the control diet (*p* < 0.05; Table 3). No significant differences were observed between treatments in heart, liver, kidney, lung, or total non-carcass component weights (*p* > 0.05). Ribeye area was numerically lower in the C + M group, although the difference was not statistically significant (*p* = 0.08).

### Meat bromatological values

Bromatological composition of the *Longissimus lumborum* is presented in Table 4 muscle of lambs fed the control or *Moringa oleifera*-supplemented diets. No significant differences were observed for dry matter, ether extract, ash, or crude protein between treatments (*P* > 0.05).

### Meat quality parameters

Cooking loss was higher in lambs fed the C + M diet than in those fed the control diet (*p* = 0.012; Table 5). Raw and cooked sample weights, shear force, thiobarbituric acid reactive substances (TBARS), chroma, hue angle, and commercial cut weights did not differ between treatments (*p* > 0.05).

## Discussion

The partial replacement of sugarcane silage with moringa preserved several qualitative carcass and meat quality traits, although lambs fed the C + M diet had lower fasting body weight and hot carcass weight. The lower fasting weight may partly reflect differences in gastrointestinal fill and body water before slaughter. However, because hot carcass weight was also reduced, differences in tissue deposition or carcass composition cannot be excluded, and this response should not be attributed solely to gastrointestinal fill. Carcass conformation and fat cover were not significantly affected by dietary treatment, indicating that external carcass finishing was broadly similar between groups. Likewise, pH measured at 45 min and 24 h postmortem did not differ between treatments, suggesting that moringa inclusion did not alter the pattern of postmortem acidification (Robles-Jiménez et al. 2022).

The lower rumen and intestinal weights observed in lambs fed the C + M diet may reflect differences in gastrointestinal contents and digesta retention associated with diet composition. However, because empty organ weights and digesta mass were not evaluated separately, this response cannot be attributed exclusively to reduced gastrointestinal fill or interpreted as evidence of structural changes in visceral tissues. These differences may have contributed to the lower fasting body weight, but they do not explain the reduction in hot carcass weight.

Meat proximate composition remained unaffected by dietary treatment, with similar dry matter, crude protein, ether extract, and ash contents between treatments. These results indicate that moringa inclusion did not measurably alter the chemical composition of lamb meat. Likewise, the evaluated color parameters did not differ between treatments, indicating no detectable effect of dietary treatment on meat color characteristics under the conditions of this study. Thiobarbituric acid reactive substances (TBARS) values also did not differ between treatments, indicating that moringa inclusion neither increased lipid oxidation nor resulted in a detectable improvement in meat oxidative stability. Although *M. oleifera* is recognized as a source of bioactive compounds with antioxidant potential, the concentrations and bioavailability of these compounds were not evaluated in direct association with meat quality in the present study. Therefore, their contribution to the observed oxidative response cannot be established.

The higher cooking loss observed in lambs fed the C + M diet suggests reduced water-holding capacity. However, because postmortem pH and meat proximate composition were similar between treatments, the mechanism underlying this response remains unclear. Unmeasured differences in muscle protein structure, myofibrillar integrity, protein denaturation, or water distribution may have contributed to the greater fluid loss during cooking (Schumacher et al. 2022). Shear force did not differ between treatments, indicating that instrumental tenderness was not affected by moringa inclusion. Nevertheless, sensory attributes and consumer acceptability were not directly evaluated and therefore cannot be inferred from the present results.

Despite the lower fasting and hot carcass weights observed at the evaluated inclusion level, moringa remains relevant as a complementary forage resource because of its nutritional and agronomic characteristics. Sugarcane silage is widely available in tropical systems but is limited by its low crude protein concentration and specific fermentative constraints (Siqueira et al. 2012; Jacovaci et al. 2017; Souza et al. 2022). In contrast, *M. oleifera* provides a protein-rich forage source, exhibits drought tolerance, produces substantial edible biomass, and can be cultivated under relatively low-input conditions (Gualberto et al. 2014; Benhammouche et al. 2021). Its known content of phenolic compounds, tannins, saponins, and other bioactive constituents also supports continued investigation of its functional potential, although these compounds cannot be directly linked to the meat quality responses observed in the present study. Therefore, moringa should not be interpreted as a universally superior or complete substitute for sugarcane silage, but rather as a complementary option capable of diversifying forage resources and improving the nutritional profile of sugarcane-based diets. Moreover, the present experiment did not include an economic analysis, and the commercial feasibility of this strategy will depend on local forage yield, establishment and harvesting costs, labor requirements, ensiling efficiency, and the availability and price of conventional protein sources.

Overall, replacing 50% of sugarcane silage with *M. oleifera* silage preserved several physicochemical meat quality traits, but it was also associated with lower fasting and hot carcass weights and greater cooking loss. Thus, moringa silage may represent a potential complementary forage resource for tropical lamb-finishing systems, although further studies are needed to determine inclusion levels that retain its nutritional and agronomic advantages while minimizing effects on carcass weight and water-holding capacity. Future research should also assess economic feasibility, sensory quality, and the amino acid and fatty acid profiles of the meat.

**Table 1:** Chemical composition of the experimental silages, concentrate, and raw forage ingredients (g/kg dry matter, except dry matter, expressed as g/kg fresh matter)

Component	C	C + M	Concentrate	Sugarcane (fresh)	M. oleifera (fresh)
Dry matter	252.90	205.20	900.10	266.00	158.40
Crude protein	26.10	75.50	160.00	26.00	223.80
Ether extract	14.20	19.40	25.00	20.00	135.00
Crude fiber	142.20	172.10	100.00	243.90	28.00
Ash	70.40	77.60	100.00	67.00	94.60
Nitrogen-free extract	747.10	655.50	-	665.00	508.80
Total digestible nutrients	-	643.40	-	683.00	622.00
Neutral detergent fiber	540.80	544.90	-	440.00	551.00
Acid detergent fiber	335.50	378.80	-	237.00	448.00
Calcium	-	-	1.30	-	-
Phosphorus	-	-	0.40	-	-
Magnesium	-	-	1.65	-	-

C = sugarcane silage; C + M = mixed silage containing sugarcane and *Moringa oleifera* at a 50:50 ratio on a dry matter basis; DM = dry matter. Fresh sugarcane and fresh *M. oleifera* represent the raw materials used for silage preparation

**Table 2:** Carcass characteristics and postmortem meat quality (pH values, temperatures and conformation)

Variable	C	C + M	*p*-value
Fasting weight (kg)	35 ± 2.12^a^	32 ± 3.51^b^	0.0092
Hot carcass weight (kg)	17.8 ± 1.45^a^	15.8 ± 1.15^b^	0.0039
Ph_0_	6.3 ± 0.61	6.3 ± 0.52	0.9943
Ph_24_	6.07 ± 0.12	6.04 ± 0.09	0.53
Conformation	2.9 ± 0.36	2.4 ± 0.65	0.06
Fat cover	3.1 ± 0.32	2.7 ± 0.64	0.12
Carcass temperature 24 h (ºC)	6.5 ± 0.94	6.6 ± 0.46	0.63
Cold carcass weight (kg)	16.8 ± 2.56	14.7 ± 2.65	0.06

Values are presented as means ± standard deviations. Means within the same row followed by different superscript letters differ according to Tukey’s test (*p* < 0.05). C = diet containing sugarcane silage; C + M = diet containing mixed sugarcane and *Moringa oleifera* silage at a 50:50 ratio on a dry matter basis

**Table 3:** Visceral organ weights and ribeye area of lambs fed sugarcane silage (C) or sugarcane + *Moringa oleifera* silage (C + M)

Variable	C	C + M	*p*-value
Rumen (kg)	5.65 ± 1.76^a^	4.22 ± 1.07^b^	< 0.001
Intestines (kg)	2.93 ± 0.41^a^	2.37 ± 0.44^b^	0.03
Heart (kg)	0.20 ± 0.04	0.20 ± 0.02	0.74
Liver (kg)	0.60 ± 0.13	0.60 ± 0.11	0.71
Kidney (kg)	0.20 ± 0.06	0.20 ± 0.03	0.11
Lungs (kg)	0.60 ± 0.17	0.60 ± 0.17	0.99
NCarc (kg)	5.55 ± 2.14	5.64 ± 2.10	0.37
Ribeye area (cm^2^)	15.00 ± 0.81	12.00 ± 1.22	0.08

Values are presented as means ± standard deviations. Means within the same row followed by different superscript letters differ according to Tukey’s test (*p* < 0.05). C = diet containing sugarcane silage; C + M = diet containing mixed sugarcane and *Moringa oleifera* silage at a 50:50 ratio on a dry matter basis; NCarc = total non-carcass component weight

**Table 4:** Proximate composition of the *Longissimus lumborum* muscle of lambs fed sugarcane silage or sugarcane and *Moringa oleifera* silages

Variable	C	C + M
DM	24.5 ± 0.31	23.87 ± 0.28
EE	1.54 ± 0.64	1.44 ± 0.68
PB	26.7 ± 0.91	28.7 ± 0.88
MM	1.31 ± 0.21	1.34 ± 0.19

Values are presented as means ± standard deviations. C = diet containing sugarcane silage; C + M = diet containing mixed sugarcane and *Moringa oleifera* silage at a 50:50 ratio on a dry matter basis

**Table 5:** Cooking traits, instrumental tenderness, lipid oxidation, color parameters, and commercial cut weights of lambs fed sugarcane silage or sugarcane and *Moringa oleifera* silages

Variable	C	C + M	*p*-value
Raw sample weight (kg)	0.055 ± 0.004	0.061 ± 0.003	0.080
Cooked sample weight (kg)	0.045 ± 0.003	0.038 ± 0.003	0.140
Cooking loss (%)	14.10 ± 1.76ᵇ	30.10 ± 6.44ᵃ	0.012
Shear force (N)	2.37 ± 0.09	2.11 ± 0.16	0.196
TBARs (mg MDA/kg)	0.22 ± 0.03	0.17 ± 0.03	0.620
Chroma (C*)	20.1 ± 0.12	20.2 ± 0.22	0.800
Hue (hº)	37.7 ± 1.34	33.71 ± 1.12	0.200
Shoulder (kg)	1.70 ± 0.42	1.50 ± 0.16	0.130
Leg (kg)	3.00 ± 0.20	2.40 ± 0.32	0.200
Neck (kg)	0.70 ± 0.21	0.70 ± 0.23	0.870
Rib (kg)	1.60 ± 0.22	1.50 ± 0.34	0.110
Loin (kg	2.00 ± 0.42	1.90 ± 0.59	0.530

Values are presented as means ± standard deviations. Means within the same row followed by different superscript letters differ according to Tukey’s test (*p* < 0.05). C = diet containing sugarcane silage; C + M = diet containing sugarcane and *Moringa oleifera* silages at a 50:50 ratio on a dry matter basis; TBARS = thiobarbituric acid reactive substances; MDA = malondialdehyde; C* = chroma; h° = hue angle

## Data Availability

The datasets generated and/or analyzed during the current study are available from the corresponding author upon request.
